# An LC/MS/MS method for analyzing the steroid metabolome with high accuracy and from small serum samples[S]

**DOI:** 10.1194/jlr.D119000591

**Published:** 2020-01-21

**Authors:** Teng-Fei Yuan, Juan Le, Shao-Ting Wang, Yan Li

**Affiliations:** Department of Clinical Laboratory, Renmin Hospital of Wuhan University, Wuhan, China

**Keywords:** hormones, derivatization, liquid chromatography tandem mass spectrometry, pregnancy, global metabolite analysis, cholesterol, steroid-related disease

## Abstract

Analyzing global steroid metabolism in humans can shed light on the etiologies of steroid-related diseases. However, existing methods require large amounts of serum and lack the evaluation of accuracy. Here, we developed an LC/MS/MS method for the simultaneous quantification of 12 steroid hormones: testosterone, pregnenolone, progesterone, androstenedione, corticosterone, 11-deoxycortisol, cortisol, 17-hydroxypregnenolone, 17-hydroxyprogesterone, dehydroepiandrosterone, estriol, and estradiol. Steroids and spiked internal standards in 100 μl serum were extracted by protein precipitation and liquid-liquid extraction. The organic phase was dried by evaporation, and isonicotinoyl chloride was added for steroid derivatization, followed by evaporation under nitrogen and redissolution in 50% methanol. Chromatographic separation was performed on a reverse-phase PFP column, and analytes were detected on a triple quadrupole mass spectrometer with ESI. The lower limits of quantification ranged from 0.005 ng/ml for estradiol to 1 ng/ml for cortisol. Apparent recoveries of steroids at high, medium, and low concentrations in quality control samples were between 86.4% and 115.0%. There were limited biases (−10.7% to 10.5%) between the measured values and the authentic values, indicating that the method has excellent reliability. An analysis of the steroid metabolome in pregnant women highlighted the applicability of the method in clinical serum samples. We conclude that the LC/MS/MS method reported here enables steroid metabolome analysis with high accuracy and reduced serum consumption, indicating that it may be a useful tool in both clinical and scientific laboratory research.

Steroid hormones are endogenous compounds that are synthesized from cholesterol by multiple enzymes in the gonads, adrenal glands, and placenta (1, 2). Although steroids are present at low concentrations, they play a vital role in human health and have been reported to be associated with many diseases, including type 2 diabetes (3), polycystic ovary syndrome (4), and depression (5). In steroid-related research, steroid metabolome analysis including steroids and their metabolites seems to be more critical than assaying a single steroid to obtain more comprehensive information (1, 6, 7). Thus, accurate steroid metabolome analysis is considered very important for exploring the mechanism of steroid-related diseases.

Traditional methods for steroid analysis are based on immunoassays (IAs) due to their accessibility and ease of use (8). However, IA techniques often lack sufficient specificity, especially when steroids are present at very low concentrations (9). The use of IAs can be also compromised by their matrix interferences, limited dynamic range, and single analyte measurement at a time (10). More MS methods have been developed for steroid detection due to their high specificity and sensitivity. GC/MS has a long history and remains an effective tool for steroid analysis; however, extremely high analytical and biochemical knowledge is needed for GC/MS steroid analysis, which is performed in a few highly specialized laboratories (11–13). An alternative and complementary analytical technique that has been used for decades is LC/MS/MS. LC/MS/MS methods can provide high analytical sensitivity and specificity and simultaneously detect multiple analytes, which exerts important roles on metabolomics studies (2, 14, 15). Considering these superiorities, many LC/MS/MS methods have been developed to measure steroid profiling or steroid metabolome. However, there are some problems that remain unresolved. One of the shortcomings of the existing methods is a lack of traceability for the steroid metabolome. Such deficient traceability inevitably will reduce the recognition of the results from these methods. Another problem is that these methods for steroid metabolome analysis cannot detect important estrogens such as estradiol and estriol in a single analysis (14, 16) because the estrogens are often detected under negative mode, which is incompatible with the other steroids (1). In addition, these methods require a large sample volume (10, 14, 17), which reduces the applicability of the method. Therefore, it is necessary to develop a new LC/MS/MS method for comprehensive steroid metabolome analysis to solve these problems.

In this study, we established a novel LC/MS/MS method based on acylation derivatization with alcoholic hydroxyl groups for the steroid metabolome, including progestogens, androgens, estrogens, mineralocorticoids, and glucocorticoids (**Fig. 1**), and this proposed method was successfully applied to detect the steroid metabolome in second-trimester pregnant women. The proposed method shows a number of advantages. First, the method was proven to exhibit high accuracy by certified reference materials covering most steroids except for pregnenolone, 17-hydroxypregnenolone, and estriol. Second, only 100 μl serum was required in this method, and the serum consumption was very low among the reported methods analyzing the steroid metabolome (1, 12, 14, 17, 18). The minimized serum consumption will increase the applicability of method. Third, the important estrogens (estradiol and estriol) were included in steroid profiling. In this proposed method, similar to other steroids, estrogens could react to generate derivative products that could be detected in the ESI positive mode. Therefore, we achieved the simultaneous quantification for estrogens and other steroids in a single analysis.

**Fig. 1.**
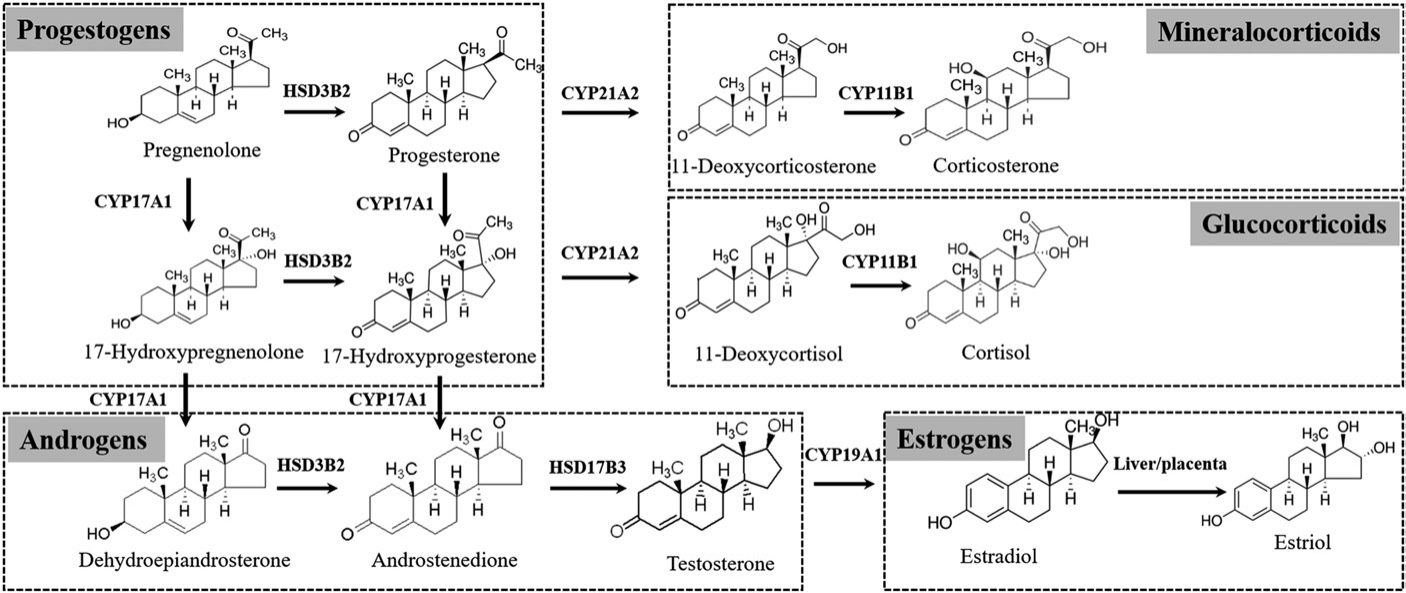
Schematic diagram of steroid metabolism.

## MATERIALS AND METHODS

### Subjects

In this study, we enrolled 30 singleton second-trimester pregnant women and analyzed their steroid metabolome using the proposed LC/MS/MS method. Informed consent was obtained from the subjects and there was protocol approval from Ethics Committee of Renmin Hospital of Wuhan University. The study abides by the Declaration of Helsinki principles.

### Chemicals and reagents

Pregnenolone (Preg), pregnenolone-17,21,21,21-d4 (Preg-d4), 17-hydroxypregnenolone (17OHPreg), 17-hydroxypregnenolone-21,21,21-d3 (17OHPreg-d3), 17-hydroxyprogesterone (17OHP), 17-hydroxyprogesterone-2,2,4,6,6,21,21,21-d8 (17OHP-d8), corticosterone (CORT), corticosterone-2,2,4,6,6,17,21,21-d8 (CORT-d8), cortisol (COR), 11-deoxycortisol (DOC), estradiol (E2), estriol (E3), estriol-2,4,17-d3 (E3-d3), and progesterone-2,2,4,6,6,17,21,21,21-d9 (P-d9) were purchased from Toronto Research Chemicals (Toronto, Canada). Cortisol-9,11,12,12-d4 (COR-d4), 11-deoxycortisol-2,2,4,6,6-d5 (DOC-d5), progesterone, estradiol-2,4-d2 (E2-d2), androstenedione-2,3,4-^13^C_3_ (AD-^13^C3), dehydroepiandrosterone-2,2,3,4,4,6-d6 (DHEA-d6), testosterone-16,16,17-d3 (T-d3), methyl tert-butyl ether (MTBE), and isonicotinoyl chloride (INC) were purchased from Sigma-Aldrich (Beijing, China). We obtained dehydroepiandrosterone (DHEA), androstenedione (AD), and testosterone from Aladdin Industrial Corporation (Shanghai, China). Dichloromethane was purchased from J&K Scientific (Beijing, China). HPLC-grade methanol and acetonitrile were purchased from Thermo Fisher Scientific (Waltham, MA). Purified water was produced using a Milli-Q apparatus (Millipore, Bedford, MA). DC Mass Spect Gold from Golden West Diagnostics (Temecula, CA) was used as a blank matrix, which was prepared by treating human serum with charcoal to get rid of steroids. We obtained SRM 971 from the National Institute of Standards and Technology and BCR 576, 577, and 578 from the Institute for Reference Materials and Measurements. MassCheck® Steroid Panel 1 Serum Control and MassCheck Steroid Panel 2 Serum Control were purchased from Chromsystems Instruments and Chemicals (Munich, Germany). We prepared an INC solution by dissolving 10 mg INC in 1 ml acetonitrile. After ultrasonic treatment and centrifugation, the supernatant was used for derivatization.

### Working solutions, calibrators, and quality control samples

We prepared stock solutions of each analyte and internal standard (IS) in acetonitrile at 1 mg/ml. Working solutions were prepared in acetonitrile containing the following steroid concentrations: E2, 40 ng/ml; E3, 2 ng/ml; testosterone, 800 ng/ml; Preg, 400 ng/ml; 17OHPreg, 800 ng/ml; 17OHP, 400 ng/ml; CORT, 2,000 ng/ml; COR, 40,000 ng/ml; DOC, 200 ng/ml; DHEA, 2,000 ng/ml; AD, 400 ng/ml; and progesterone, 400 ng/ml. We prepared IS working solutions containing the following concentrations: E2-d2, 4 ng/ml; E3-d3, 200 ng/ml; T-d3, 80 ng/ml; Preg-d4, 40 ng/ml; 17OHPreg-d3, 80 ng/ml; 17OHP-d8, 40 ng/ml; CORT-d8, 200 ng/ml; COR-d4, 2,000 ng/ml; DOC-d5, 20 ng/ml; DHEA-d6, 200 ng/ml; AD-^13^C3, 40 ng/ml; and P-d9, 40 ng/ml. We prepared six calibrators at different concentrations (C1–C6) covering physiological ranges by adding working solutions into the blank matrix followed by stepwise dilution using the blank matrix. The concentrations of steroids in calibrators are listed in supplemental Table S1. The quality control (QC) samples were prepared at three levels by adding working solutions into the blank matrix (high, medium, and low QC, respectively).

### Sample preparation

We transferred 100 μl of calibrators, QC samples, and serum samples to polypropylene tubes. Then, 5 μl of IS working solution and 200 μl acetonitrile were added, followed by vortexing (30 s) for protein precipitation. Subsequently, 1 ml MTBE was added, and the mixture was vortexed for 5 min for liquid-liquid extraction. After centrifugation (12,000 rpm, 5 min), the upper organic layer was transferred to a clean tube and evaporated under nitrogen at 55°C. The dried residue was redissolved in 100 μl dichloromethane and 10 μl INC. The final mixture was then evaporated under nitrogen at 55°C and reconstituted in 100 μl 50% methanol and transferred to an autosampler vial.

### LC/MS/MS instrumentation and analytical conditions

The LC instrumentation was an LC-20AD chromatograph (Shimadzu, Kyoto, Japan), and the tandem mass spectrometer was an AB SCIEX 4500 QTRAP mass spectrometer. We conducted the chromatographic separation of steroids on a Kinetex™ 2.6 μm PFP 100 Å column (100 × 3 mm) with a flow rate of 0.3 ml/min. The column temperature was set to 45°C, and the injection volume was 20 μl. Mobile phases were purified water (A) and methanol (B). The gradient elution was summarized as follows: 0–2 min, 60% B; 2–13.9 min, 60% to 100% B; 13.9–14.0 min, 60% B; and 14–21 min, 60% B. The total analysis required 21 min, and more polar compounds were diverted to waste for the first 6.8 min to keep the mass spectrometer clean.

The mass spectrometer was operated in positive mode using an ESI source. The analytes were monitored by multiple reaction monitoring, and optimized quantitative mass transitions are listed in **Table 1**. In addition, positive ESI mass spectra of derivatized steroids are shown in supplemental Figs. S1–S3). The cleavages and fragments of analytes are shown in supplemental Table S2. Data acquisition and processing were conducted using AB SCIEX Analyst version 1.6.2.

**TABLE 1.** Multiple reaction monitoring parameters for steroids using LC/MS/MS

Analytes	Precursor Ion	Product Ion	DP (V)	CE (eV)
E2-IN	378.3	124.1	90	25
E2-d2-IN	380.3	124.0	90	25
E3-IN	394.1	124.0	100	25
E3-d3-IN	397.3	124.0	70	30
T-IN	394.2	124.0	90	30
T-d3-IN	397.2	123.9	90	32
Preg-IN	422.3	124.0	100	95
Preg-d4-IN	426.3	124.0	80	25
17OHPreg-IN	438.4	124.0	80	35
17OHPreg-d3-IN	441.4	123.9	80	30
17OHP-IN	436.2	79.1	100	80
17OHP-d8-IN	444.2	79.0	100	80
CORT-IN	452.3	434.1	120	30
CORT-d8-IN	460.3	442.3	100	30
COR-IN	468.2	124.1	80	35
COR-d4-IN	472.4	124.0	100	35
DOC-IN	452.3	123.9	80	40
DOC-d5-IN	457.3	124.1	80	40
DHEA-IN	394.3	124.0	80	25
DHEA-d6-IN	400.3	124.0	80	25
AD	287.2	97.0	100	30
AD-13C3	290.2	100	100	30
Progesterone	315.2	97.1	90	23
P-d9	324.1	100.1	100	28

CE, collision energy; DP, declustering potential.

### Method validation

To validate the LC/MS/MS method, we evaluated the accuracy, linearity, sensitivity, selectivity, precision, matrix effect, and stability in accordance with the US Food and Drug Administration and Clinical and Laboratory Standards document C62-A.

To assess accuracy, we calculated apparent recoveries by dividing the measured steroid concentrations to the nominal spiked values in the blank matrix, and the values of apparent recoveries should be between 85% and 115%. The calibration curves were calculated by measuring the six calibrators in triplicate using a linear least-squares regression model, and the coefficient of determination (*R*^2^) was used to evaluate the linearity. The lower limits of quantification (LLOQs) were defined as the lowest concentration in serum that could be quantitatively measured with imprecision (<20%) and accuracy (within ±20%). To evaluate selectivity, we respectively spiked high concentrations (C6) of 12 steroids into low-QC and high-QC samples to fabricate interference-introduced serum, and then we calculated the apparent recoveries of steroids except for the introduced steroid, and the apparent recoveries should be between 85% and 115%. Method imprecision was assessed using QC samples at three concentrations. The measurement was performed six times in one day (intra-assay) and in six consecutive days (interassay). The CV of measurement values was used to represent the imprecision, and the CV should not be greater than 15%. Matrix effects (MEs) were calculated with the equation *ME* = *B*/*A*, where *B* represents the peak area ratios of steroids to ISs from extracts after liquid-liquid extraction of the matrix, and *A* refers to the peak area ratios of steroids to ISs from extracts after liquid-liquid extraction of pure water. The ME values should be between 85% and 115%. Stability experiments were performed using QC samples at three levels. First, storage stability was assessed after storage at room temperature (12 h and 72 h), 4°C (72 h and 7 d), and −80°C (30 d and 60 d). Second, freeze-thaw stability was tested after three cycles of freezing (−80°C) and thawing (25°C). Third, posttreatment stability was evaluated at 4°C and 25°C for 4 h and 24 h.

Moreover, we evaluated the traceability of this method using certified reference materials. The MassCheck Steroid Panel included testosterone, E2, progesterone, AD, DHEA, 17OHP, COR, CORT, and DOC at three concentrations. The SRM included testosterone, progesterone, and COR at two concentrations, and the BCR included E2 at three concentrations. These reference materials were measured in triplicate using the proposed method, and then the measured values were compared with the certified values.

## RESULTS AND DISCUSSION

### The selection of the extracting solvents

**Fig. 2.**
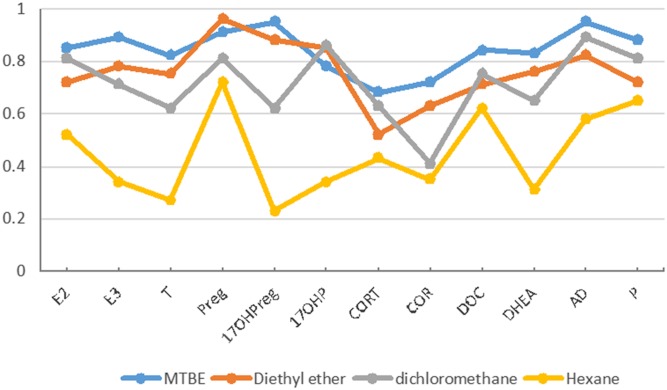
The extraction efficiency of four organic solvents (MTBE, diethyl ether, dichloromethane, and hexane) for steroids.

After the protein precipitation, liquid-liquid extraction was conducted. In order to obtain maximum extraction efficiency, we compared the extraction recoveries of several organic solvents, including MTBE, diethyl ether, dichloromethane, and hexane. Extraction recoveries were evaluated on the basis of two groups. For group A, a standard solution mixture of steroids was added to blank serum before extraction, and the IS mixture was added to the organic extract after extraction. For group B, both the standard and IS mixture were added after extraction. Extraction recoveries were calculated by dividing the peak areas ratios of steroids to the IS in group A to the ratios in group B. As shown in **Fig. 2**, MTBE produced the best extraction recovery for most steroids. Therefore, MTBE was chosen as the extracting solvent for liquid-liquid extraction in this method.

### Optimization of the derivatization reaction conditions

**Fig. 3.**
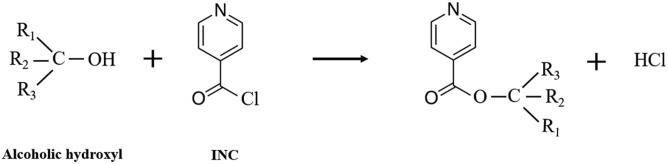
Derivatization reaction scheme for steroids containing alcoholic hydroxyl groups with INC.

The derivatization reaction is shown in **Fig. 3**. To achieve the greatest reaction efficiency, several parameters were optimized, including reaction solvents, time, and temperature. We found that dichloromethane was the most conducive to the reaction compared with ACN, methylbenzene, ethyl acetate, hexane, MTBE, and trichloromethane. Moreover, evaporation by nitrogen after adding dichloromethane and INC was an indispensable step for the reaction. The reason may be that evaporation could lead to the volatilization of HCl and then facilitate the reaction. Before evaporation, time and temperature could have little influence on the reaction. Considering the stability of steroids at high temperatures and pretreatment efficiency, immediate evaporation after introducing dichloromethane and INC and a temperature of 55°C were chosen. The entire evaporation required 2 min. Consequently, the reaction was allowed to complete within 2 min. There are two main advantages of isonicotinoyl ester derivatization compared with other derivatization methods. First, this derivatization reaction is simple and fast. It takes just 2 min. Second, sodium and other nonvolatile substances or trifluoroacetic acid would be not introduced. These substances may influence the ionization or damage the chromatographic column and mass spectrometer.

### Method validation

#### Sensitivity and linearity.

The LLOQ and linearity for steroids are listed in **Table 2**. Such sensitivity was sufficient, even for special groups of individuals such as children and postmenopausal women, and the *R*^2^ values for the linearity of steroids were not less than 0.99. Although AD and progesterone were detected without derivatization due to the lack of alcoholic hydroxyl groups, their sensitivity was not compromised by the process of derivatization.

**TABLE 2.** Linearity and LLOQ for steroids

Analytes	Retention Time (min)	Calibration Range (ng/ml)	Linear Regression (*R*^2^)	LLOQ (ng/ml)
E2	11.88	0.01-0.4	*y* = 1.8705*x* + 0.0345 (0.99)	0.005
E3	8.56	0.5-20	*y* = 0.0876*x* + 0.0083 (0.99)	0.1
Testosterone	12.60	0.2-8	*y* = 0.6287*x* − 0.009 (0.99)	0.05
Preg	14.13	0.1-4	*y* = 0.0859*x* + 0.0152 (0.99)	0.05
17OHPreg	12.43	0.2-8	*y* = 0.2828*x* + 0.0199 (0.99)	0.1
17OHP	12.00	0.1-4	*y* = 1.0856*x* − 0.0831 (0.99)	0.05
CORT	9.26	0.5-20	*y* = 0.1523*x* + 0.0389 (0.99)	0.2
COR	7.55	10-400	*y* = 0.0059*x* + 0.0423 (0.99)	1
DOC	10.06	0.05-2	*y* = 3.4378*x* − 0.1282 (0.99)	0.05
DHEA	12.81	0.5-20	*y* = 0.0857*x* + 0.0357 (0.99)	0.2
AD	8.07	0.1-4	*y* = 0.2082*x* + 0.0101 (0.99)	0.01
Progesterone	10.51	1-40	*y* = 3.4962*x* + 0.4218 (0.99)	0.01

#### Selectivity.

Due to the similar structures of steroids, we evaluated the specificity by introducing steroids as analog interferences of other steroids. After introducing interferences, we found no significant effect for targets’ apparent recoveries (ranging from 86.5% to 102.8%), indicating satisfactory selectivity of this method.

#### Accuracy and imprecision.

Both accuracy and imprecision were evaluated on three QC levels: high, medium, and low. Apparent recoveries of steroids at high-, medium- and low-QC concentrations are listed in **Table 3**. For all steroids, the apparent recoveries were between 86.4% and 115.0% at different concentrations. After evaluating method precision, we found that intra-assay and interassay precisions were between 5.3% and 12.8% for each steroid (**Table 4**). These results demonstrated satisfactory accuracy and precision.

**TABLE 3.** Concentrations of steroids in low-QC, medium-QC, and high-QC samples and apparent recoveries of steroids

Analytes	Low QC		Medium QC		High QC	
	Concentrations (ng/ml)	Recovery (%)	Concentrations (ng/ml)	Recovery (%)	Concentrations (ng/ml)	Recovery (%)
E2	0.02	99.2	0.08	99.5	0.2	99.4
E3	1	97.7	4	106.6	10	101.3
Testosterone	0.4	99.8	1.6	102.3	4	97.6
Preg	0.2	92.5	0.8	106.9	2	95.6
17OHPreg	0.2	95.8	0.8	115.0	2	104.0
17OHP	0.2	109.2	0.8	103.1	2	90.4
CORT	1	86.4	4	98.3	10	95.8
COR	20	109.9	80	112.3	200	106.4
DOC	0.1	96.3	0.4	98.6	1	101.0
DHEA	1	89.6	4	109.0	10	98.3
AD	0.2	98.3	0.8	108.0	2	105.0
Progesterone	0.2	89.1	0.8	114.1	2	108.6

**TABLE 4.** Intra- and interassay precisions of steroid metabolome analysis

	Low QC		Medium QC		High QC	
	Intra-assay	Interassay	Intra-assay	Interassay	Intra-assay	Interassay
Analytes	*%*					
E2	7.6	10.5	10.6	8.9	9.7	10.2
E3	8.9	11.2	9.2	10.5	8.2	11.8
Testosterone	12.3	9.6	8.7	9.2	10.6	10.9
Preg	9.8	8.2	6.9	8.6	9.5	12.6
17OHPreg	7.2	10.5	5.3	10.3	8.9	9.5
17OHP	10.1	8.1	7.8	11.5	7.9	8.7
CORT	12.8	7.9	6.5	9.9	10.5	9.2
COR	6.5	10.2	7.9	10.2	8.3	10.8
DOC	8.8	11.1	10.5	12.1	9.7	9.1
DHEA	9.6	8.2	7.3	8.6	8.2	10.6
AD	9.5	9.3	8.1	10.8	9.9	12.3
Progesterone	8.3	8.1	7.9	9.6	10.3	11.2

#### Matrix effect and stability.

The ME ranges of steroids for all concentration levels were all between 91.8% and 103.5%, indicating that there was no significant ion suppression or enhancement from the matrix, and the quantification of steroids would not be affected by the serum matrix during steroid analysis. Although the commercial blank matrix is widely used for matrix effect evaluation, we should recognize the possibility that the charcoal-stripped process could remove many other components that themselves may affect matrix effect.

After conducting stability experiments, we found that all steroids could be well preserved in serum for 72 h at 25°C, 7 d at 4°C, and 60 d at −80°C, and the changes in steroid concentrations were within 10% of theoretical concentrations after three freeze-thaw cycles. Posttreatment stability experiments showed that the final products were stable for 24 h at 4°C and 25°C.

### Traceability from certified reference materials

After measuring the certified reference materials and comparing the measured values with certified values at different levels, we found that there were limited biases (−10.7% to 10.5%) between the measured values of our developed method and the certified values in the SRM and BCR reference materials (**Table 5**) and the MassCheck Steroid Panel Serum Control (**Table 6**). These results show the high accuracy of our methods.

**TABLE 5.** Certified and measured values of steroids in the SRM and BCR reference materials

Analytes	Levels	Certified Value	Measured Value (*n*** = **3)	Bias	CV
		*ng/ml*		*%*	
E2	BCR 576	0.031	0.028 ± 0.001	−9.7	3.6
	BCR 577	0.188	0.171 ± 0.006	−9.0	3.3
	BCR 578	0.364	0.381 ± 0.015	4.6	3.8
Testosterone	SRM 971F	0.277	0.264 ± 0.014	−4.8	5.1
	SRM 971M	6.425	6.186 ± 0.338	−3.7	5.5
Progesterone	SRM 971F	1.946	1.828 ± 0.180	−6.0	9.8
	SRM 971M	0.041	0.037 ± 0.002	−9.8	2.7
COR	SRM 971F	90.786	82.822 ± 3.056	−8.7	3.69
	SRM 971M	107.738	98.977 ± 3.398	−8.1	3.43

**TABLE 6.** Certified and measured values of steroids in the MassCheck® Steroid Panel Serum Control

Analytes	Levels	Certified Value	Measured Value (*n* = 3)	Bias	CV
		*ng/ml*		*%*	
Testosterone	Level 1	0.202	0.192 ± 0.013	−5.1	7.0
	Level 2	1.51	1.450 ± 0.089	−3.9	6.1
	Level 3	7.85	7.703 ± 0.199	−1.9	2.6
E2	Level 1	0.084	0.075 ± 0.006	−10.3	7.3
	Level 2	0.425	0.392 ± 0.013	−7.7	3.4
	Level 3	2.63	2.452 ± 0.075	−6.8	3.0
Progesterone	Level 1	0.352	0.314 ± 0.023	−10.7	7.2
	Level 2	3.24	3.051 ± 0.113	−5.8	3.7
	Level 3	15.9	16.720 ± 1.031	5.2	6.2
17OHP	Level 1	0.304	0.314 ± 0.033	3.3	10.5
	Level 2	1.52	1.457 ± 0.093	−4.2	6.4
	Level 3	9.18	8.530 ± 0.425	−7.1	5.0
COR	Level 1	25.1	23.153 ± 1.569	−7.8	6.8
	Level 2	59.8	55.91 ± 2.95	−6.5	5.3
	Level 3	175	168.5 ± 11.5	−3.7	6.8
CORT	Level 1	0.797	0.722 ± 0.043	−9.4	6.0
	Level 2	3.98	3.767 ± 0.316	−5.4	8.4
	Level 3	28.7	26.26 ± 2.26	−8.5	8.6
DOC	Level 1	0.286	0.307 ± 0.010	7.5	3.3
	Level 2	1.44	1.353 ± 0.040	−6.0	3.0
	Level 3	9.40	8.787 ± 0.198	−6.5	2.26
DHEA	Level 1	2.13	2.193 ± 0.131	2.9	5.9
	Level 2	12.4	11.437 ± 1.072	−7.8	9.4
	Level 3	41.0	38.20 ± 1.25	−6.8	3.3
AD	Level 1	0.315	0.33 ± 0.01	4.8	3.0
	Level 2	1.26	1.198 ± 0.033	−4.9	2.78
	Level 3	10.3	9.63 ± 0.31	−6.5	3.3

### Clinical application

**Fig. 4.**
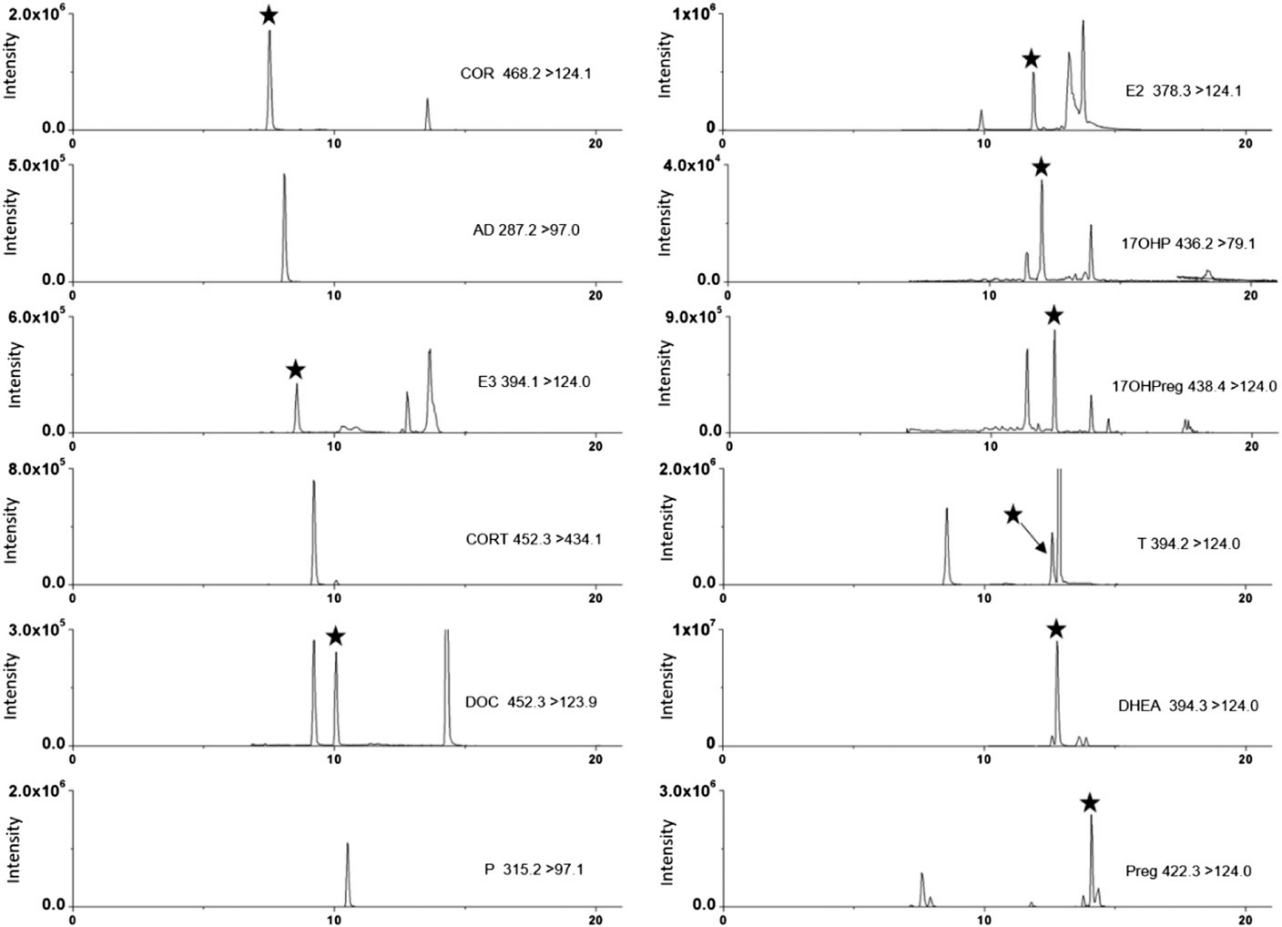
Chromatographic separation of steroids in serum samples from pregnant women, including the respective *m/z* ratio of the quantifier ion. Asterisks indicate the target peak.

Pregnancy is a special period for women. During this period, steroids are of particular importance for normal pregnancy, and the concentrations of many steroids undergo great changes. In this study, we enrolled 30 singleton second-trimester pregnant women and analyzed their steroid metabolome using the proposed LC/MS/MS method. The separation efficiency remained excellent during real sample detection, and chromatograms of steroids in serum samples of pregnant women are shown in **Fig. 4**. The baseline characteristics and steroid concentrations are listed in **Table 7**.

**TABLE 7.** Baseline characteristics and steroid concentrations of pregnant women

Variables	Mean ± SD	Range
Ages (years)	31.68 ± 4.95	24–40
Gestational age (weeks)	17.26 ± 2.46	12.43–22.86
Steroids (ng/ml)		
Testosterone	0.56 ± 0.17	0.32–0.98
E2	4.64 ± 1.37	2.30–7.64
DHEA	6.37 ± 3.29	2.01–14.01
COR	143.34 ± 49.86	55.68–233.59
CORT	3.71 ± 3.35	0.37–11.66
E3	1.09 ± 0.73	0.17–3.58
Preg	2.01 ± 0.56	1.10–3.57
17OHPreg	0.24 ± 0.19	0.03–0.75
17OHP	2.60 ± 1.05	1.35–6.09
AD	1.74 ± 1.42	0.31–7.66
Progesterone	67.61 ± 15.12	30.50–95.04
DOC	0.82 ± 0.35	0.41–1.94

## CONCLUSION

In summary, this LC/MS/MS method describes a reliable and sensitive assay based on acylation derivatization for steroid metabolome that possesses high accuracy and requires very low serum consumption. This method was successfully applied in a steroid metabolome analysis of pregnant women and is a promising avenue for future research on steroid metabonomics.

## Supplementary Material

Supplemental Data
