# Supplementary material for: An LC/MS/MS method for analyzing the steroid metabolome with high accuracy and from small serum samples
Source: J Lipid Res. 2020 Jan 21;61(4):580–6. doi: 10.1194/jlr.D119000591 (PMC7112139; doi:10.1194/jlr.D119000591)
Supplement: Supplemental Data [file supp_61_4_580__index.html]

An LC-MS/MS method to analyze the steroid metabolome with high accuracy and from small serum samples — An LC-MS/MS method to analyze steroid metabolome — An LC/MS/MS method for analyzing the steroid metabolome with high accuracy and from small serum samples — Supplemental Data 

# An LC/MS/MS method for analyzing the steroid metabolome with high accuracy and from small serum samples

## Supplemental Data

- Supplementary - supporting information
