# Supplementary material for: An LC/MS/MS method for analyzing the steroid metabolome with high accuracy and from small serum samples
Source: J Lipid Res. 2020 Jan 21;61(4):580–6. doi: 10.1194/jlr.D119000591 (PMC7112139; doi:10.1194/jlr.D119000591)
Supplement: Supplemental Data [file supp_D119000591_157846_2_supp_460744_q49mr4.doc]

**Supplementary Materials**

**An LC-MS/MS method to analyze the steroid metabolome with high accuracy and from small serum samples**

Teng-Fei Yuan (袁腾飞)^†^, Juan Le (乐娟)^†^, Shao-Ting Wang (王少亭)*****, Yan Li (李艳)*****

Department of Clinical Laboratory, Renmin Hospital of Wuhan University, Wuhan 430060, Hubei Province, China.

^†^These authors contributed equally to this work.

*These authors jointly supervised this work.

*Correspondence: Shao-Ting Wang (shaotingw@163.com); Yan Li (liyanlcms@163.com; Tel: +86-27-88041911)

**Supplemental Tables**

**Table S1**. The concentrations of steroids in calibrators.

| Analytes | Calibrator concentrations (ng/mL) | | | | | |
| --- | --- | --- | --- | --- | --- | --- |
|  | C1 | C2 | C3 | C4 | C5 | C6 |
| E2 | 0.01 | 0.02 | 0.04 | 0.08 | 0.2 | 0.4 |
| E3 | 0.5 | 1 | 2 | 4 | 10 | 20 |
| T | 0.2 | 0.4 | 0.8 | 1.6 | 4 | 8 |
| Preg | 0.1 | 0.2 | 0.4 | 0.8 | 2 | 4 |
| 17OHPreg | 0.1 | 0.2 | 0.4 | 0.8 | 2 | 4 |
| 17OHP | 0.1 | 0.2 | 0.4 | 0.8 | 2 | 4 |
| CORT | 0.5 | 1 | 2 | 4 | 10 | 20 |
| COR | 10 | 20 | 40 | 80 | 200 | 400 |
| DOC | 0.05 | 0.1 | 0.2 | 0.4 | 1 | 2 |
| DHEA | 0.5 | 1 | 2 | 4 | 10 | 20 |
| AD | 0.1 | 0.2 | 0.4 | 0.8 | 2 | 4 |
| P | 0.1 | 0.2 | 0.4 | 0.8 | 2 | 4 |

E2, estradiol; E3, estriol; T, testosterone; Preg, pregnenolone; P, progesterone; AD, androstenedione; 17OHPreg, 17-hydroxypregnenolone; 17OHP, 17-hydroxyprogesterone; CORT, corticosterone; DOC, 11-deoxycortisol; COR, cortisol; DHEA, dehydroepiandrosterone.

**Table S2**. The cleavages and fragments of analytes.

| Analytes | Cleavages and fragments |
| --- | --- |
| E2, E2-d2, E3, E3-d3, T, T-d3, Preg, Preg-d4, 17OHPreg, 17OHPreg-d3, COR, COR-d4, DOC, DOC-d5, DHEA, DHEA-d5  (M/M-d→124) | 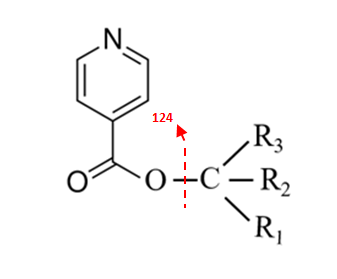 |
| AD  (287.2 → 97.0) | 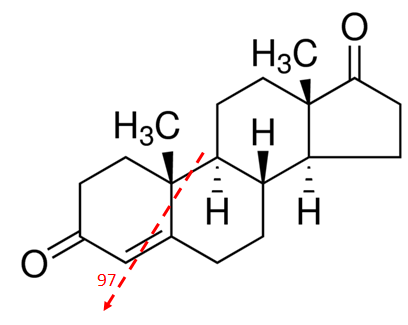 |
| AD-^13^C_3_  (290.2 → 100.0) | 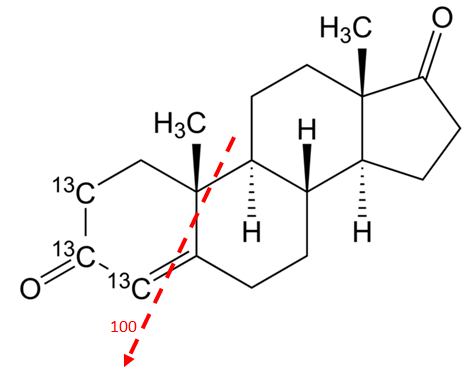 |
| P  (315.2 → 97.1) | 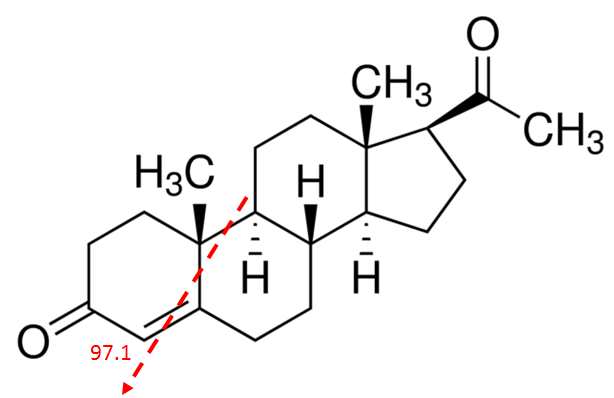 |
| P-d9  (324.1 → 100.1) | 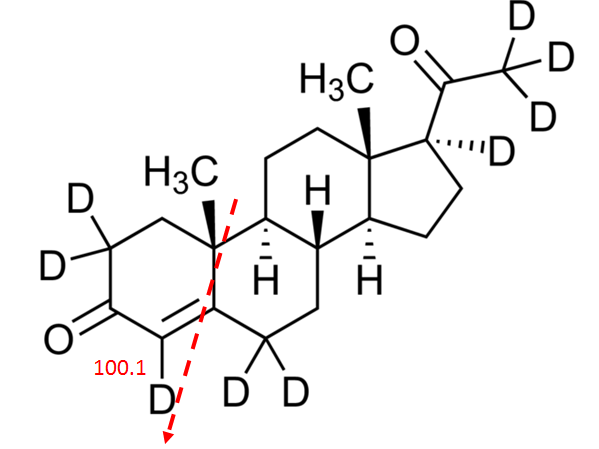 |
| 17OHP/17OHP-d8  (M/M-d → 79.1) | 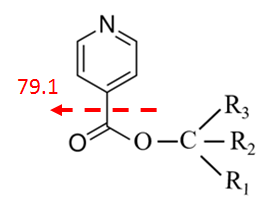 |
| CORT*  (452.3 → 434.1) | 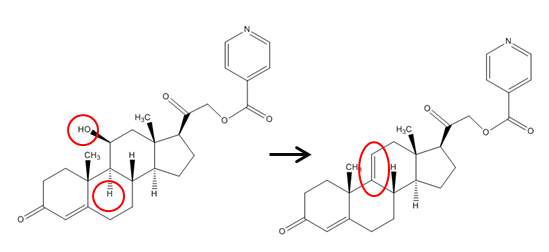 |
| CORT-d8*  (460.3 → 442.3) | 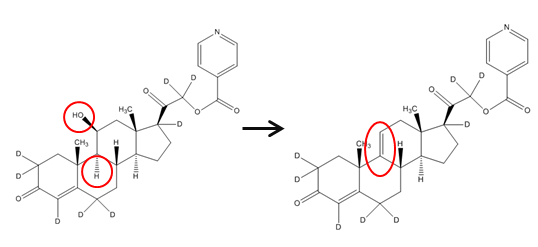 |

*CORT and CORT-d8 lost H_2_O and then were ionized.

**Supplemental Figures**


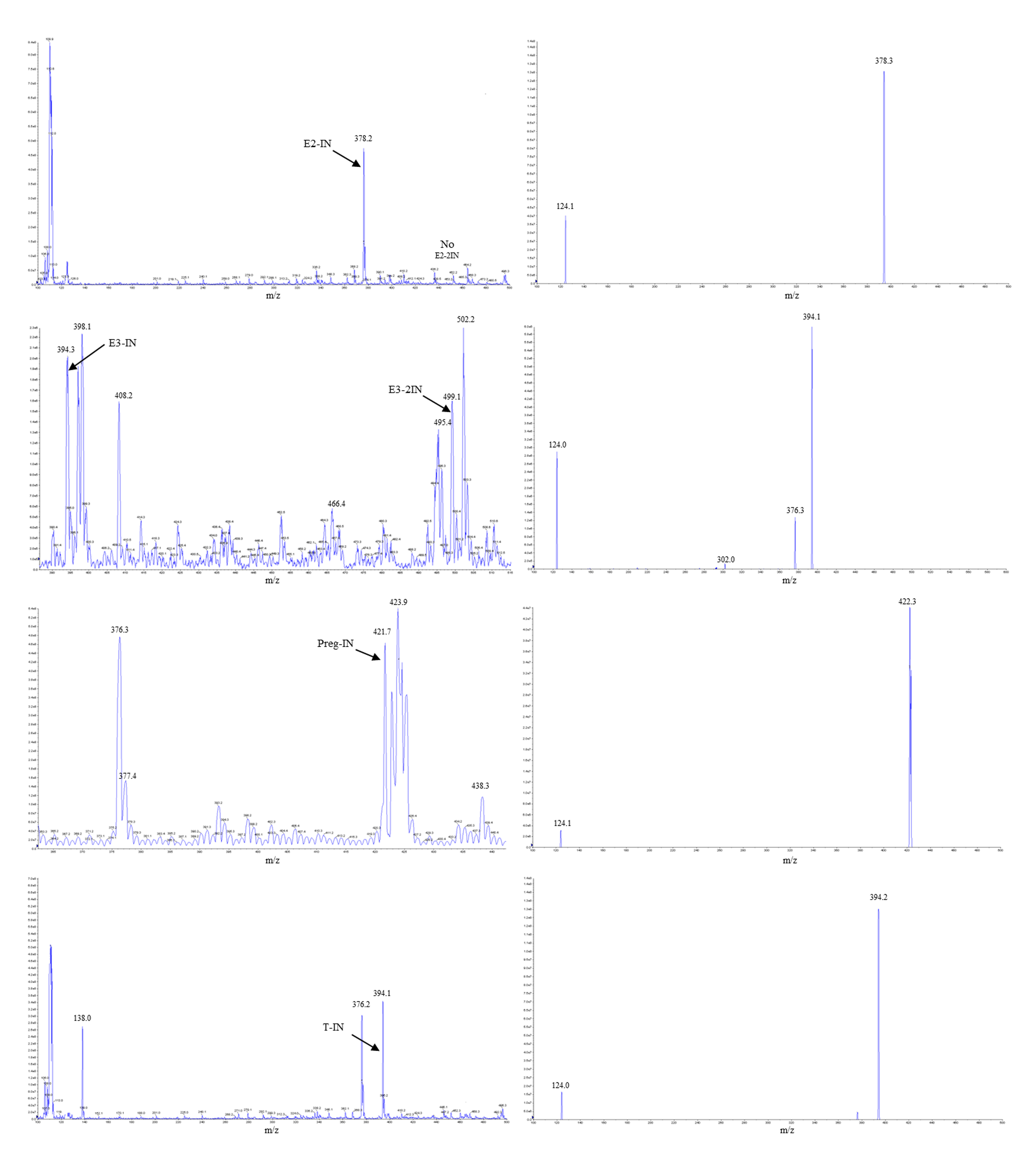
**Figure S1**. Positive mass spectra of derivative products of E2, E3, Preg and T. (The right of the figure represents the mass spectra of Q1 scan mode and the left represents the mass spectra of Product Ion scan mode)


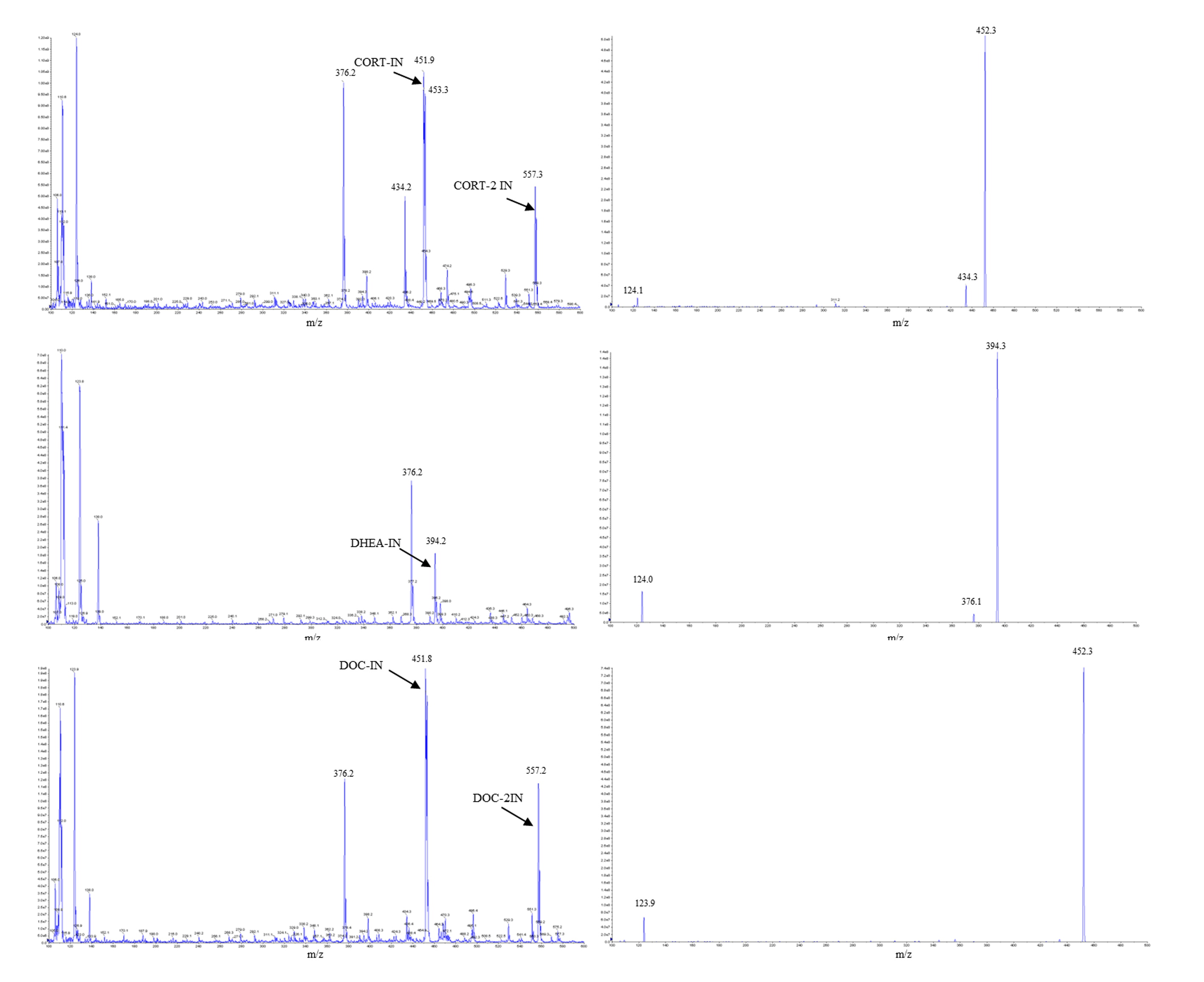


**Figure S2**. Positive mass spectra of derivative products of CORT, DHEA and DOC. (The right of the figure represents the mass spectra of Q1 scan mode and the left represents the mass spectra of Product Ion scan mode)


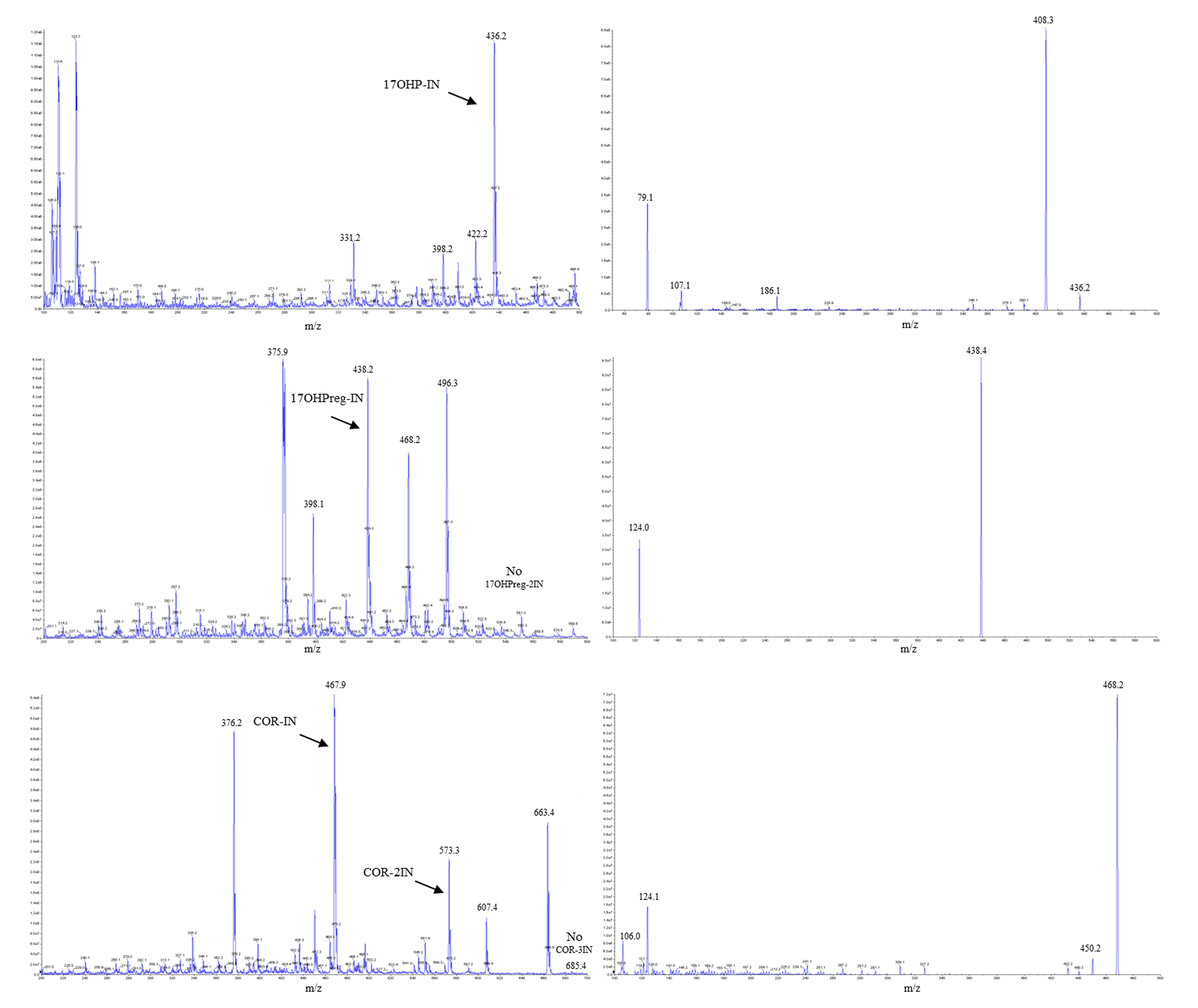


**Figure S3**. Positive mass spectra of derivative products of 17OHP, 17OHPreg and COR. (The right of the figure represents the mass spectra of Q1 scan mode and the left represents the mass spectra of Product Ion scan mode)
